# Animal Magnetism

**Published:** 1886-07

**Authors:** 


### HALL'S JOURNAL OF HEALTH

Vol. 33. JULY, 1886. No. 7.

### ANIMAL MAGNETISM.

In the June number of the JOURNAL, the writer treated of Mesmerism, but necessarily in a cursory manner, the object of that and similar brief articles being merely to introduce to our readers such important discoveries relating to life and health, as have stood the test of time, and the scrutiny of able and experienced investigators, leaving only the barriers of despotic ignorance to be overcome, to give them place among the revealed treasures of science, to which the world owes in great measure, its material progress.

Without animal magnetism there could be no such thing as mesmerism, as it is by the influence of this subtle element, whatever it may be that the mesmerist is able to bring the mind of his subject under control.

Like its sister science it met with little else than derisive opposition upon its introduction to public notice. A popular work published by a leading New York house, as recently as 1856, under the suggestive title of "The World's Progress," classes "Animal Magnetism," among the noted deceptions of its period, with no higher rating than the schemes of John Law and the South Sea bubble.

Indeed for more than a century, this was the popular belief concerning a discovery in which the common mind refused to acquiesce or even entertain with any degree of patience.

The theory advanced by Mesmer, at Vienna, in 1766, that magnetism is a subtle element which permeates all bodies and forms of life, and is capable of being exerted and intensified by the human will, has never been successfully controverted. It may be compared to those essential elements of life, air and water, which are present wherever life exists, and made subject to the extraordinary needs of mankind by the efforts of genius and the appliances of art. That magnetism *is*, all know who care to know, but *what* it is, we are as incapable of explaining as of defining what electricity is, whose omnipresence is manifested in so many ways.

Webster defines magnetism as "a supposed agent of a peculiar and mysterious nature said to have a powerful influence on the patient when acted upon by contact, or voluntary emotion on the part of the operator," and refers to mesmerism in corelation.

But animal magnetism, and mesmerism are no more identical than steam and the steam engine, or water and the hydraulic engine. The former is always at hand to be exerted in an extraordinary manner, through the will and person of the mesmerist, and the *fact of mesmerism* implies this human instrumentality, as hydraulic force systematically exerted, implies the use of *artificial* appliances. The existence of the one is a self evident *fact*, call it what you will ; the other is the *factor* through whose volition its powers are concentrated and actively employed for special ends, for instance to counteract pain or cure disease.

Animal magnetism is *natural* and *normal* but the mesmeric sleep is *abnormal*, and though the former may be imparted by one person to another involuntarily and quite unintentionally; when thus imparted, it is not mesmerism because it is not accomplished by, or subjected to the will of an operator, being rather an unconscious out-flowing of a superfluity of vitality on the one part, to supply a deficiency on the other, even as water of different degrees of temperament, will interpermeate and become equalized.

There is no question but that there are numerous persons so generously endowed with this vitalizing fluid, as to be capable of alleviating pain and healing disease, by physical contact, who have no conception of it themselves We have been made acquainted with a number of instances of cure by such healers without contiguity or manipulation of any sort, their mere presence being sufficient to effect that object. This is particularly the case in respect to the lighter forms of nervous troubles. The presence of such persons in the sick chamber is of itself a healing balm to the afflicted, who is able to feel the vitalizing force though ignorant of its source. On the other hand there are persons whose presence at the bed-side of a sufferer only adds to his suffering. Hence it is that the family physician, of all others, should be naturally refined and sympathetic, at once capable of comprehending not alone the physical, but also the mental or spiritual wants of his patients, and in a manner, ministering to them out of his abundant sympathy and good cheer.

It is however, a mistake to suppose that a magnetizer however powerful his influence with the general run of patients, can be equally successful with all persons. He may fail entirely in many instances, without any fault or lack of power upon his part, simply because the magnetic elements brought into requisition, are either inharmonious or too much alike. Hence it is that kinsmen, and those holding marital relations are frequently incapable of imparting to one another the vitalizing force which a stranger of a suitable temperament, would accomplish with hardly an effort. This feature is not sufficiently appreciated, even among those who practice magnetic healing as a calling. If more generally recognized and honestly lived up to, they would in such cases stand aside, or assist the patient to a fitter means of recovery.

Cahagnet, an able French writer upon this subject, in a work published more tnan thirty years ago, gives an account in detail of a large number of experiments with subjects brought under the influence of magnetism, including some remarkable cures effected either directly or by means of information thus obtained; cases indeed, which were abandoned as incurable by ordinary methods, and submitted to magnetic treatment as a *dernier resort,* and more recent authors are equally positive in their facts. These evidences of magnetic cures coming down to us in a broadening march of more than two centuries, are of a most convincing kind. Are the stiff necks of the medical profession never to bend in acknowledgement of truths like these, sacred to health? Is it now either too late or too early to mend? Are they determined to go on, forcing their patients to die regularly, rather than permit them to be cured irregularly? They make *their* appeal to Legislatures; we make *ours* to the heads and hearts of intelligent communities.

